# High genetic diversity and geographic subdivision of three lance nematode species (*Hoplolaimus* spp.) in the United States

**DOI:** 10.1002/ece3.1568

**Published:** 2015-07-03

**Authors:** Claudia M Holguin, Juan A Baeza, John D Mueller, Paula Agudelo

**Affiliations:** 1 Department of Agricultural and Environmental Sciences, Clemson UniversityClemson, South Carolina, 29634; 2 Department of Biological Sciences, Clemson UniversityClemson, South Carolina, 29634; 3 Edisto Research and Education Center, Clemson UniversityBlackville, South Carolina, 29817

**Keywords:** Barcoding, genetic variability, *Hoplolaimus* spp., lance nematodes, phylogeny

## Abstract

Lance nematodes (*Hoplolaimus* spp.) feed on the roots of a wide range of plants, some of which are agronomic crops. Morphometric values of amphimictic lance nematode species overlap considerably, and useful morphological characters for their discrimination require high magnification and significant diagnostic time. Given their morphological similarity, these *Hoplolaimus* species provide an interesting model to investigate hidden diversity in crop agroecosystems. In this scenario, *H. galeatus* may have been over-reported and the related species that are morphologically similar could be more widespread in the United States that has been recognized thus far. The main objectives of this study were to delimit *Hoplolaimus galeatus* and morphologically similar species using morphology, phylogeny, and a barcoding approach, and to estimate the genetic diversity and population structure of the species found. Molecular analyses were performed using sequences of the cytochrome c oxidase subunit 1 (*Cox*1) and the internal transcribed spacer (ITS1) on 23 populations. Four morphospecies were identified: *H. galeatus, H. magnistylus, H. concaudajuvencus,* and *H. stephanus,* along with a currently undescribed species. Pronounced genetic structure correlated with geographic origin was found for all species, except for *H. galeatus*. *Hoplolaimus galeatus* also exhibited low genetic diversity and the shortest genetic distances among populations. In contrast, *H. stephanus*, the species with the fewest reports from agricultural soils, was the most common and diverse species found. Results of this project may lead to better delimitation of lance nematode species in the United States by contributing to the understanding the diversity within this group.

## Introduction

Lance nematodes (*Hoplolaimus* spp.) are relatively long and robust vermiform nematodes with a distinct cephalic region and massive well-developed stylets (Sher 1963; Fortuner 1991) that feed on the roots of a diversity of monocotyledonous and dicotyledonous plants. *Hoplolaimus* species reported in the southeastern United States include *H. columbus* Sher, 1963; *H. galeatus* (Cobb, 1913) Thorne, 1935, *H. magnistylus* Robbins, 1982; *H. stephanus* Sher, 1963; *H. seinhorsti* Luc, 1958, and *H. tylenchiformis* von Daday, 1905 (Lewis and Fassuliotis, 1982). *Hoplolaimus columbus*, *H. galeatus*, and *H. magnistylus* are considered to be economically important and can cause serious damage to agronomic crops, including cotton (*Gossypium hirsutum* L.), corn (*Zea mays* L.) and soybean (*Glycine max* L.) (Fassuliotis, 1974; Nyczepir and Lewis, 1979; Robbins et al., 1987, 1989; Henn and Dunn, 1989; Noe, 1993). Ma et al. (2011) suggest that a fourth species, *H. stephanus*, may be of economic importance on grasses.

**Figure 1:**
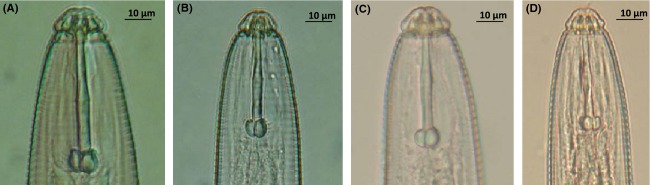
Head regions of the four *Hoplolaimus* species collected in this study. (A) *H. magnistylus,* (B) *H. galeatus,* (C) *H. concaudajuvencus, and* (D) *H. stephanus*.

Of the twenty-nine species described in the genus (Handoo and Golden 1992), *H. galeatus* is the most commonly reported in the United States (Lewis and Fassuliotis 1982). Morphologically, *Hoplolaimus galeatus* belongs to a group of species that Fortuner (1991) called “ancestral”, characterized by three esophageal gland nuclei, four incisures in the lateral field, excretory pore posterior to the hemizonid, and the presence of abundant males. Siddiqi (2000) classifies this group as the subgenus *Hoplolaimus*. This “ancestral” subgenus includes *H. magnistylus, H. concaudajuvencus,* and *H. stephanus*, among a few other species. *Hoplolaimus magnistylus* and *H. concaudajuvencus* differ from *H. galeatus* by the possession of a longer stylet (Table1, Fig.1). *Hoplolaimus concaudajuvencus* has more definitely tulip-shaped stylet knobs and a second-stage juvenile with conically pointed tail. *Hoplolaimus stephanus* can be distinguished from *H*. *galeatus* by the 24–28 longitudinal striations on the basal annule of the lip region compared to 32–36 in *H. galeatus*, shorter spicules, less areolation of the lateral field, and shorter body length (Sher 1963) (Table1, Fig.1). The “derived” species, which constitute the subgenera *Basirolaimus* and *Ethiolaimus* and include species such as *H. columbus* and *H. seinhorsti*, possess six esophageal gland nuclei and fewer than four incisures in the lateral field, the excretory pore is anterior to the hemizonid, and the mode of reproduction is mostly parthenogenetic, with males rare or absent (Fortuner 1991).

**Table 1:** Published data for morphological characters and morphometrics of *Hoplolaimus concaudajuvencus, Hoplolaimus galeatus, Hoplolaimus magnistylus, and Hoplolaimus stephanus*

Species, (source), and (number of specimens)	Length (mm)	Lateral incisures	Esophageal gland nuclei	Stylet length (*μ*m)	Labial annules	Longitudinal striae on basal lip annule	Position of excretory pore	Phasmids in relation to vulva	Tail annules	Spicule length (*μ*m)
*H. concaudajuvencus* (Golden and Minton 1970) (*n* = 20 ♀, 20 ♂)	1.12–2.04	4	3	50.4–56.6	5–6	36	Posterior to hemizonid	1 anterior	7–14	45.0–56.0
								1 posterior		
*H. galeatus* (Sher 1963) (*n* = 20 ♀, 10 ♂)	1.24–1.94	4	3	43.0–52.0	5	32–36	Posterior to hemizonid	1 anterior	10–16	40.0–52.0
								1 posterior		
*H. magnistylus* (Robbins 1982) (*n* = 30 ♀, 20 ♂)	1.36–1.97	4	3	52.0–61.0	4–6	22–340	Posterior to hemizonid	1 anterior	12–17	52.0–58.0
								1 posterior		
*H. stephanus* (Sher 1963) (*n* = 20 ♀, 10 ♂)	1.01–1.45	4	3	43.0–50.0	4	24–28	Posterior to hemizonid	1 anterior	12	30.0–38.0
								1 posterior		

Morphometric values of the “ancestral” species overlap considerably (Table1, Fig.1) (Sher 1963; Vovlas et al. 1991; Handoo and Golden 1992), and useful morphological characters for their discrimination require high magnification and significant diagnostic time. Given their morphological similarity, these *Hoplolaimus* species within the “ancestral” clade provide an interesting model to investigate hidden diversity in crop agroecosystems, considering the wide host range and distribution of *H. galeatus* in the United States (Fortuner 1991). In this scenario, *H. galeatus* may have been over-reported and the related species within the subgenus *Hoplolaimus* (Siddiqi 2000) that are morphologically similar could be more widespread in the United States that has been recognized thus far. The main objectives of this study were (1) to delimit *Hoplolaimus galeatus* and morphologically similar species using morphology, phylogeny, and a barcoding approach and (2) to estimate the genetic diversity and population structure of the species found. With this study, we expect to understand the diversity within this group and contribute to the elucidation of the delimitation of species of lance nematodes in the United States.

## Materials and Methods

### Nematode sampling, identification, and DNA isolation

Nematode populations were obtained from soil samples collected in 2011–2013 in agricultural fields, golf courses, and lawns of different regions in the United States (Table2). Nematodes were extracted from soil by sugar centrifugal flotation (Jenkins 1964). Lance nematode specimens from each soil sample were morphologically identified using the key by Handoo and Golden (1992) and the original descriptions of the species (Sher 1963; Golden and Minton 1970; Robbins 1982). DNA from individual nematodes was extracted using the Sigma Extract-N-Amp kit (XNAT2) (Sigma, St. Louis, MO) as reported by Ma et al. (2011), and DNA was stored at −20°C until use.

**Table 2:** Specimen sample information and GenBank accession numbers of *Hoplolaimus* species used in this study

Morphospecies	Location	Host	Host	Specimen	Accession number	Accession number
		Scientific name	Common name	code	COI	ITS
*H. stephanus*	Tyrell County, NC	*Glycine max*	Soybean	NCS32-4	KP230605	–
*H. stephanus*	Tyrell County, NC	*Glycine max*	Soybean	NCS32-5	KP230606	KP303652
*H. stephanus*	Tyrell County, NC	*Glycine max*	Soybean	NCS32-7	KP230607	KP303653
*H. stephanus*	Tyrell County, NC	*Glycine max*	Soybean	NCS32-8	KP230608	–
*H. stephanus*	Tyrell County, NC	*Glycine max*	Soybean	NCS32-12	KP230609	–
*H. stephanus*	Tyrell County, NC	*Glycine max*	Soybean	NCS32-13	KP230610	KP303654
*H. stephanus*	Tyrell County, NC	*Glycine max*	Soybean	NCS32-15	KP230611	KP303655
*H. stephanus*	Tyrell County, NC	*Glycine max*	Soybean	NCS32-17	–	KP303656
*H. stephanus*	Washington County, NC	*Glycine max*	Soybean	NCS35-4	–	KP303657
*H. stephanus*	Washington County, NC	*Glycine max*	Soybean	NCS35-5	KP230612	–
*H. stephanus*	Washington County, NC	*Glycine max*	Soybean	NCS35-6	KP230613	–
*H. stephanus*	Washington County, NC	*Glycine max*	Soybean	NCS35-7	KP230614	–
*H. stephanus*	Washington County, NC	*Glycine max*	Soybean	NCS35-8	KP230615	KP303658
*H. stephanus*	Washington County, NC	*Glycine max*	Soybean	NCS35-11	KP230616	KP303659
*H. stephanus*	Bertie County, NC	*Glycine max*	Soybean	NCS7-3	KP230596	–
*H. stephanus*	Bertie County, NC	*Glycine max*	Soybean	NCS7-5	KP230597	–
*H. stephanus*	Bertie County, NC	*Glycine max*	Soybean	NCS7-8	KP230598	–
*H. stephanus*	Bertie County, NC	*Glycine max*	Soybean	NCS7-9	KP230599	KP303647
*H. stephanus*	Bertie County, NC	*Glycine max*	Soybean	NCS7-13	–	KP303648
*H. stephanus*	Camden County, NC	*Glycine max*	Soybean	NCS10-5	–	KP303649
*H. stephanus*	Camden County, NC	*Glycine max*	Soybean	NCS10-7	KP230600	–
*H. stephanus*	Camden County, NC	*Glycine max*	Soybean	NCS10-8	KP230601	–
*H. stephanus*	Camden County, NC	*Glycine max*	Soybean	NCS10-9	–	KP303650
*H. stephanus*	Camden County, NC	*Glycine max*	Soybean	NCS10-10	KP230602	–
*H. stephanus*	Camden County, NC	*Glycine max*	Soybean	NCS10-12	–	KP303651
*H. stephanus*	Camden County, NC	*Glycine max*	Soybean	NCS10-13	KP230603	–
*H. stephanus*	Camden County, NC	*Glycine max*	Soybean	NCS10-15	KP230604	–
*H. stephanus*	Story County, IA	*Zea mays*	Corn	IAC1-1	KP230568	KP303614
*H. stephanus*	Story County, IA	*Zea mays*	Corn	IAC1-2	KP230569	KP303615
*H. stephanus*	Story County, IA	*Zea mays*	Corn	IAC1-3	KP230570	KP303616
*H. stephanus*	Story County, IA	*Zea mays*	Corn	IAC1-4	KP230571	KP303617
*H. stephanus*	Holt County, NE	*Zea mays*	Corn	NEC9-1	KP230617	–
*H. stephanus*	Holt County, NE	*Zea mays*	Corn	NEC9-2	–	KP303660
*H. stephanus*	Holt County, NE	*Zea mays*	Corn	NEC9-3	KP230618	–
*H. stephanus*	Holt County, NE	*Zea mays*	Corn	NEC9-4	KP230619	KP303661
*H. stephanus*	Holt County, NE	*Zea mays*	Corn	NEC9-5	KP230620	–
*H. stephanus*	Holt County, NE	*Zea mays*	Corn	NEC9-6	KP230621	–
*H. stephanus*	Holt County, NE	*Zea mays*	Corn	NEC9-10	KP230622	KP303662
*H. stephanus*	Holt County, NE	*Zea mays*	Corn	NEC9-11	KP230623	–
*H. stephanus*	Holt County, NE	*Zea mays*	Corn	NEC9-13	KP230624	KP303663
*H. stephanus*	Holt County, NE	*Zea mays*	Corn	NEC9-15	KP230625	–
*H. stephanus*	Riley County, KS	*Agrostis* sp.	Bentgrass	KSB-1	KP230589	KP303640
*H. stephanus*	Riley County, KS	*Agrostis* sp.	Bentgrass	KSB-2	KP230590	KP303641
*H. stephanus*	Riley County, KS	*Agrostis* sp.	Bentgrass	KSB-3	KP230591	KP303642
*H. stephanus*	Riley County, KS	*Agrostis* sp.	Bentgrass	KSB-4	KP230594	KP303643
*H. stephanus*	Riley County, KS	*Agrostis* sp.	Bentgrass	KSB-5	KP230595	KP303644
*H. stephanus*	Riley County, KS	*Agrostis* sp.	Bentgrass	KSB-6	KP230592	KP303645
*H. stephanus*	Riley County, KS	*Agrostis* sp.	Bentgrass	KSB-7	KP230593	KP303646
*H. stephanus*	Warren County, OH	*Agrostis* sp.	Bentgrass	OHB1-2	KP230626	KP303664
*H. stephanus*	Warren County, OH	*Agrostis sp*	Bentgrass	OHB1-4	–	KP303665
*H. stephanus*	Warren County, OH	*Agrostis* sp.	Bentgrass	OHB1-5	KP230627	–
*H. stephanus*	Warren County, OH	*Agrostis* sp.	Bentgrass	OHB1-7	KP230628	KP303666
*H. stephanus*	Warren County, OH	*Agrostis* sp.	Bentgrass	OHB1-8	KP230629	–
*H. stephanus*	Warren County, OH	*Agrostis* sp.	Bentgrass	OHB1-9	KP230630	KP303667
*H. stephanus*	Warren County, OH	*Agrostis* sp.	Bentgrass	OHB1-10	KP230631	–
*H. stephanus*	Warren County, OH	*Agrostis* sp.	Bentgrass	OHB1-12	KP230632	KP303668
*H. stephanus*	Warren County, OH	*Agrostis* sp.	Bentgrass	OHB1-13	KP230633	KP303669
*H. stephanus*	Warren County, OH	*Agrostis* sp.	Bentgrass	OHB1-14	KP230634	–
*H. stephanus*	Warren County, OH	*Agrostis* sp.	Bentgrass	OHB1-15	KP230635	KP303670
*H. stephanus*	Warren County, OH	*Agrostis* sp.	Bentgrass	OHB1-16	KP230636	KP303671
*H. magnistylus*	Weakley County, TN	*Zea mays*	Corn	TNC1-4	–	KP303675
*H. magnistylus*	Weakley County, TN	*Zea mays*	Corn	TNC1-7	KP230653	–
*H. magnistylus*	Weakley County, TN	*Zea mays*	Corn	TNC1-8	–	KP303678
*H. magnistylus*	Weakley County, TN	*Zea mays*	Corn	TNC1-9	KP230654	–
*H. magnistylus*	Weakley County, TN	*Zea mays*	Corn	TNC2-1	KP230655	KP303677
*H. magnistylus*	Weakley County, TN	*Zea mays*	Corn	TNC2-2	–	KP303678
*H. magnistylus*	Weakley County, TN	*Zea mays*	Corn	TNC2-3	KP230656	KP303679
*H. magnistylus*	Weakley County, TN	*Zea mays*	Corn	TNC2-4	–	KP303680
*H. magnistylus*	Weakley County, TN	*Zea mays*	Corn	TNC2-5	KP230657	KP303681
*H. magnistylus*	Weakley County, TN	*Zea mays*	Corn	TNC2-6	–	KP303682
*H. magnistylus*	Massac County, IL	*Glycine max*	Soybean	ILS4-2	KP230572	KP303618
*H. magnistylus*	Massac County, IL	*Glycine max*	Soybean	ILS4-3	KP230573	KP303619
*H. magnistylus*	Massac County, IL	*Glycine max*	Soybean	ILS4-4	KP230574	KP303620
*H. magnistylus*	Massac County, IL	*Glycine max*	Soybean	ILS4-5	KP230575	KP303621
*H. magnistylus*	Massac County, IL	*Glycine max*	Soybean	ILS4-6	KP230576	KP303622
*H. magnistylus*	Massac County, IL	*Glycine max*	Soybean	ILS4-7	KP230577	KP303623
*H. magnistylus*	Massac County, IL	*Glycine max*	Soybean	ILS4-8	KP230578	KP303624
*H. magnistylus*	Massac County, IL	*Glycine max*	Soybean	ILS5-1	–	KP303625
*H. magnistylus*	Massac County, IL	*Glycine max*	Soybean	ILS5-2	KP230579	–
*H. magnistylus*	Massac County, IL	*Glycine max*	Soybean	ILS5-3	KP230580	–
*H. magnistylus*	Massac County, IL	*Glycine max*	Soybean	ILS5-4	KP230581	KP303626
*H. magnistylus*	Massac County, IL	*Glycine max*	Soybean	ILS5-5	KP230582	–
*H. magnistylus*	Massac County, IL	*Glycine max*	Soybean	ILS5-6	–	KP303627
*H. magnistylus*	Massac County, IL	*Glycine max*	Soybean	ILS5-7	KP230583	KP303628
*H. magnistylus*	Massac County, IL	*Glycine max*	Soybean	ILS5-8	KP230584	KP303629
*H. magnistylus*	Massac County, IL	*Glycine max*	Soybean	ILS5-9	KP230585	–
*H. magnistylus*	Massac County, IL	*Glycine max*	Soybean	ILS6-1	–	KP303630
*H. magnistylus*	Massac County, IL	*Glycine max*	Soybean	ILS6-2	–	KP303631
*H. magnistylus*	Massac County, IL	*Glycine max*	Soybean	ILS6-3	KP230586	KP303632
*H. magnistylus*	Massac County, IL	*Glycine max*	Soybean	ILS6-4	KP230587	KP303633
*H. magnistylus*	Massac County, IL	*Glycine max*	Soybean	ILS6-5	KP230588	KP303634
*H. magnistylus*	Massac County, IL	*Glycine max*	Soybean	ILS6-6	–	KP303635
*H. magnistylus*	Massac County, IL	*Glycine max*	Soybean	ILS6-7	–	KP303636
*H. magnistylus*	Massac County, IL	*Glycine max*	Soybean	ILS6-8	–	KP303637
*H. galeatus*	Charleston County, SC	*Agrostis* sp.	Bentgrass	SCB1-1	KP230637	–
*H. galeatus*	Charleston County, SC	*Agrostis* sp.	Bentgrass	SCB1-2	KP230638	–
*H. galeatus*	Charleston County, SC	*Agrostis* sp.	Bentgrass	SCB1-3	KP230639	–
*H. galeatus*	Charleston County, SC	*Agrostis* sp.	Bentgrass	SCB1-4	KP230640	KP303672
*H. galeatus*	Charleston County, SC	*Agrostis* sp.	Bentgrass	SCB1-5	KP230641	–
*H. galeatus*	Charleston County, SC	*Agrostis* sp.	Bentgrass	SCB1-8	KP230642	KP303673
*H. galeatus*	Charleston County, SC	*Agrostis* sp.	Bentgrass	SCB1-9	KP230643	–
*H. galeatus*	Charleston County, SC	*Agrostis* sp.	Bentgrass	SCB1-10	–	KP303674
*H. galeatus*	Horry County, SC	*Cynodon dactylon*	Bermuda	SCB2-1	KP230644	–
*H. galeatus*	Horry County, SC	*Cynodon dactylon*	Bermuda	SCB2-2	KP230645	–
*H. galeatus*	Horry County, SC	*Cynodon dactylon*	Bermuda	SCB2-3	KP230646	–
*H. galeatus*	Horry County, SC	*Cynodon dactylon*	Bermuda	SCB2-4	KP230647	–
*H. galeatus*	Horry County, SC	*Cynodon dactylon*	Bermuda	SCB2-5	KP230648	–
*H. galeatus*	Horry County, SC	*Cynodon dactylon*	Bermuda	SCB2-6	KP230649	–
*H. galeatus*	Horry County, SC	*Cynodon dactylon*	Bermuda	SCB2-7	KP230650	–
*H. galeatus*	Horry County, SC	*Cynodon dactylon*	Bermuda	SCB2-8	KP230651	–
*H. galeatus*	Horry County, SC	*Cynodon dactylon*	Bermuda	SCB2-9	KP230652	–
*H. galeatus*	St. Johns, FL	*Stenotaphrum secundatum*	St. Augustine	FLS1-2	KP230562	–
*H. galeatus*	St. Johns, FL	*Stenotaphrum secundatum*	St. Augustine	FLS1-4	KP230563	–
*H. galeatus*	St. Johns, FL	*Stenotaphrum secundatum*	St. Augustine	FLS1-5	KP230564	KP303607
*H. galeatus*	St. Johns, FL	*Stenotaphrum secundatum*	St. Augustine	FLS1-13	–	KP303608
*H. galeatus*	St. Johns, FL	*Stenotaphrum secundatum*	St. Augustine	FLS1-14	–	KP303609
*H. galeatus*	St. Johns, FL	*Stenotaphrum secundatum*	St. Augustine	FLS1-15	–	KP303610
*H. galeatus*	St. Johns, FL	*Stenotaphrum secundatum*	St. Augustine	FLS1-17	–	KP303611
*H. galeatus*	St. Johns, FL	*Stenotaphrum secundatum*	St. Augustine	FLS1-18	–	KP303612
*H. galeatus*	St. Johns, FL	*Stenotaphrum secundatum*	St. Augustine	FLS1-22	–	KP303613
*H. galeatus*	Escambia County, FL	*Agrostis* sp.	Bentgrass	FLB1-1	KP230557	KP303598
*H. galeatus*	Escambia County, FL	*Agrostis sp*	Bentgrass	FLB1-2	–	KP303599
*H. galeatus*	Escambia County, FL	*Agrostis* sp.	Bentgrass	FLB1-3	KP230558	KP303600
*H. galeatus*	Escambia County, FL	*Agrostis* sp.	Bentgrass	FLB1-4	KP230559	–
*H. galeatus*	Escambia County, FL	*Agrostis* sp.	Bentgrass	FLB1-6	–	KP303601
*H. galeatus*	Escambia County, FL	*Agrostis* sp.	Bentgrass	FLB1-7	–	KP303602
*H. galeatus*	Escambia County, FL	*Agrostis* sp.	Bentgrass	FLB1-8	KP230560	–
*H. galeatus*	Escambia County, FL	*Agrostis* sp.	Bentgrass	FLB1-9	KP230561	–
*H. galeatus*	Polk County, FL	*Stenotaphrum secundatum*	St. Augustine	FLB2-2	–	KP303603
*H. galeatus*	Polk County, FL	*Stenotaphrum secundatum*	St. Augustine	FLB2-11	–	KP303604
*H. galeatus*	Polk County, FL	*Stenotaphrum secundatum*	St. Augustine	FLB2-13	–	KP303605
*H. galeatus*	Polk County, FL	*Stenotaphrum secundatum*	St. Augustine	FLB2-19	–	KP303606
*H. galeatus*	Baldwin County, AL	*Cynodon dactylon*	Bermuda	ALB1-1	–	KP303595
*H. galeatus*	Baldwin County, AL	*Cynodon dactylon*	Bermuda	ALB1-2	KP230554	KP303596
*H. galeatus*	Baldwin County, AL	*Cynodon dactylon*	Bermuda	ALB1-8	KP230555	–
*H. galeatus*	Baldwin County, AL	*Cynodon dactylon*	Bermuda	ALB1-9	KP230556	KP303597
*H*. sp.	Dallas County, TX	*Agrostis* sp.	Bentgrass	TXB1-1	KP230660	–
*H*. sp.	Dallas County, TX	*Agrostis* sp.	Bentgrass	TXB1-2	KP230661	–
*H*. sp.	Dallas County, TX	*Agrostis* sp.	Bentgrass	TXB1-3	KP230662	–
*H*. sp.	Dallas County, TX	*Agrostis* sp.	Bentgrass	TXB1-4	KP230663	–
*H*. sp.	Dallas County, TX	*Agrostis* sp.	Bentgrass	TXB1-5	KP230664	–
*H*. sp.	Dallas County, TX	*Agrostis* sp.	Bentgrass	TXB1-6	KP230665	–
*H*. sp.	Dallas County, TX	*Agrostis* sp.	Bentgrass	TXB2-2	KP230666	–
*H*. sp.	Dallas County, TX	*Agrostis* sp.	Bentgrass	TXB2-3	KP230667	KP303685
*H*. sp.	Dallas County, TX	*Agrostis* sp.	Bentgrass	TXB2-9	KP230668	KP303686
*H*. sp.	Dallas County, TX	*Agrostis* sp.	Bentgrass	TXB2-12	KP230669	–
*H*. sp.	Dallas County, TX	*Agrostis* sp.	Bentgrass	TXB2-13	KP230670	–
*H*. sp.	Dallas County, TX	*Agrostis* sp.	Bentgrass	TXB2-19	KP230671	–
*H*. sp. 1	Sevier County, TN	*Acer* sp.	Maple	TNM-1	–	KP303683
*H*. sp. 1	Sevier County, TN	*Acer* sp.	Maple	TNM-4	KP230658	–
*H*. sp. 1	Sevier County, TN	*Acer* sp.	Maple	TNM-5	KP230659	KP303684
*H. columbus*	Tift County, GA	*Glycine max*	Soybean	GA1-1	KP230565	–
*H. columbus*	Tift County, GA	*Glycine max*	Soybean	GA1-2	KP230566	–
*H. columbus*	Tift County, GA	*Glycine max*	Soybean	GA1-3	KP230567	KP303638
*H. columbus*	Scotland County, NC	*Glycine max*	Soybean	NC85-2	–	KP303639

### PCR amplification and sequencing

The ITS region was amplified using forward primer Hoc-1f (5′-AACCTGCTGCTGGATCATTA-3′) and reverse primers: Hoc-2r (5′-CCGAGTGATCCACCGATAA-3′) (Bae et al. 2008) and LSUD-3r (5′ TATGCTTAAGTTCAGCGGGT-3′) (Bae et al. 2009), which amplify the ITS1 and the entire ITS region, respectively. Amplification from some individuals failed with the set of primers abovementioned, and new primers were designed based on sequence alignments of *H. columbus, H. stephanus, H. magnistylus,* and *H. galeatus* obtained during this study (Table2). These newly developed primers were F1-F (5′-CTGACGACCAGTTAGGCGTT-3′), F1-R (5′-CGTGCCAAAGGATGTCACTC-3′), F5-F (5′-CTTGATTGGAAAGCGCCCAC-3′), and F5-R (5′-ATGTCACTCCAATGGCGCA-3′). For the COI gene, a partial sequence was amplified using specific primers that have been successfully used for phylogenetic and population structure studies of several marine and plant-parasitic nematodes (Derycke et al. 2007; De Oliveira et al. 2012), as well as for DNA barcoding of free-living marine nematodes (Derycke et al. 2010). The forward primer was JB3 (5′ TTTTTTGGGCATCCTGAGGTTTAT 3′) (Hu et al. 2002) and the reverse primer was JB5 (5′ AGCACCTAAACTTAAAACATAATGAAA 3′) (Derycke et al. 2005). For all primers, PCRs were performed in 20 *μ*L final volume reaction, adding 8 *μ*L PCR-grade water, 10 *μ*L of ReadyMix Taq PCR Mix with MgCl_2_ (Sigma, St. Louis, MO) (20 mmol/L Tris–HCl pH 8.3, 100 mmol/L KCl, 3 mmol/L MgCl2, 0.002% gelatin, 0.4 mmol/L dNTP mixture (dATP, dCTP, dGTP, and dTTP), and 0.06 units of Taq DNA polymerase/mL), 0.5 *μ*L of each primer (20 *μ*mol/L), and 1 *μ*L of DNA template. Thermal cycling conditions for the ITS marker included: initial denaturation at 95°C for 3 min, 33 cycles of 95°C for 45 sec, 59°C for 1 min 15 sec, 72°C for 2 min, and final extension at 72°C for 10 min. For the COI portion, the initial denaturation was set at 95°C for 3 min, followed by 33 cycles of 95°C for 45 sec, 50°C for 1 min 15 sec, 72°C for 2 min, and final extension at 72°C for 10 min. The amplified products were loaded onto a 1.5% agarose gel and visualized using GelRed™ (Biotium, San Francisco, California, United States.). PCR products were purified using magnetic beads and sequenced in both directions with the ABI 3730 capillary sequencer (Applied Biosystems) in the DNA Laboratory (School of Life Sciences) at Arizona State University.

### Sequence alignment

Contigs were assembled in Sequencher 5.1 (Genes code corp., Ann Arbor, MI). All sequences were checked and edited manually, and chromatograms were inspected to confirm base calling and to identify recombination sites. Consensus DNA sequences were then aligned using ClustalW (Thompson et al. 1997) including three out-group taxa: *H. columbus* sequences obtained from this study and sequences of *Rotylenchus robustus* (JX015440) and *Rotylenchus paravitis* (JX015415) from GenBank. The original alignment for ITS consisted of 1050 bp, but several indels were detected. Therefore, divergent and ambiguously aligned positions were removed and conserved blocks selected using the software Gblocks v0.91b (Castresana 2000) with default values. The resulting dataset comprised 550 bp of the ITS1 portion of the gene. For the mitochondrial region, the alignment consisted of 347 bp. The new generated haplotypes for both genes were deposited in GenBank (Table1).

For the COI marker, the aligned sequences were well defined and chromatograms had no double peaks, ambiguous positions or indels. Nonetheless, we tested for the occurrence of stop codons that could denote the presence of nuclear copies of mitochondrial-derived genes (numts) or COI pseudogenes (Zhang and Hewitt 1996; Song et al. 2008; Moulton et al. 2010). Numts are copies of mitochondrial genes moved to the nuclear genome that become nonfunctional and noncoding. Consequently, these numts can confuse phylogenetic analyses (Song et al. 2008; Moulton et al. 2010; Baeza and Fuentes 2013). To check for the presence of numts, we followed Song et al. (2008) and did a basic local alignment search (blast) of all COI sequences in NCBI (National Center for Biotechnology Information) against the database nucleotide collection (nr/nt) and optimized for highly similar sequences to include only haplotypes that showed *E-*values ≥ 1.0e-45 and similarity ≥ 90% with plant-parasitic nematodes. All retrieved sequences were of plant-parasitic nematodes most commonly of the genera *Rotylenchus* and *Scutellonema* followed by *Heterodera, Punctodera,* and *Meloidogyne*. After this, the COI haplotypes were translated using the invertebrate mitochondrial code in Mega v.5 (Tamura et al. 2011) to verify the protein coding frameshifts and nonsense codons for each of the six putative reading frames in DNAsp (Librado and Rozas 2009).

### Phylogenetic analysis

For phylogenetic analysis, the most appropriate evolutionary model was selected for each gene dataset using the Akaike information criterion (AIC) in the software Modeltest v3.7 (Posada and Crandall 1998). For both genes, the best-fit model was GTR with invgamma-shaped rate variation (G) (0.7653 for COI and 2.1690 for ITS), and a proportion of invariable sites (I) (0.4489 for COI and 0.4330 for ITS), with nucleotide frequencies of A = 0.289, C = 0.064, G = 0.1788, T = 0.4686 for COI; and A = 0.2798, C = 0.2886, G = 0.2163, T = 0.2153 for ITS. Phylogenetic relationships were constructed for each gene separately using maximum-likelihood (ML) and Bayesian inference (BI). Maximum-likelihood analysis was performed in Treefinder (Gangolf et al. 2004) using the default parameters. Branch support was based on 1000 bootstrap pseudoreplicates (Felsenstein 1985), and clades were considered as well/strongly supported when bootstrap was >70%. Bayesian inference was implemented in the software MrBayes 3.1.2 (Ronquist and Huelsenbeck 2003). The analysis was conducted for 6 million generations, and trees were sampled for every 100th generation from the Markov Monte Carlo chain (MCMC) analysis. A burn-in period was set to discard the first 1250 trees with nodal support defined as posterior probabilities, and clades were considered strongly supported when values were > 0.95 (Alfaro et al. 2003). Additionally, for the COI sequences, a neighbor-joining (NJ) tree was constructed in MEGA v.5.0 (Tamura et al. 2011) using the Kimura two-parameter (K2P) model under the default settings. From this dataset, a pairwise distance matrix among haplotypes was generated to calculate intra- and interspecific genetic divergence among haplotypes, as it is typically performed for DNA barcoding studies (Hebert et al. 2004).

### Genetic diversity

Genetic diversity analyses were based on mitochondrial DNA data because COI gene trees provided better resolution than ITS1. Mitochondrial haplotypes networks were constructed for each species using median-joining (MJ) networks in Network 4.5.1.0 (http://www.fluxus-engineering.com/sharenet.htm). Analysis of molecular variance (AMOVA) was conducted in Arlequin v. 3.11 (Excoffier et al. 2005) to infer genetic structure within species. Values of Fst were also calculated in Arlequin v. 3.11 to estimate genetic differentiation among clades and tested for significance by permuting haplotypes between species/populations (10,000 replicates).

## Results

### Phylogenetic relationships

**Figure 2:**
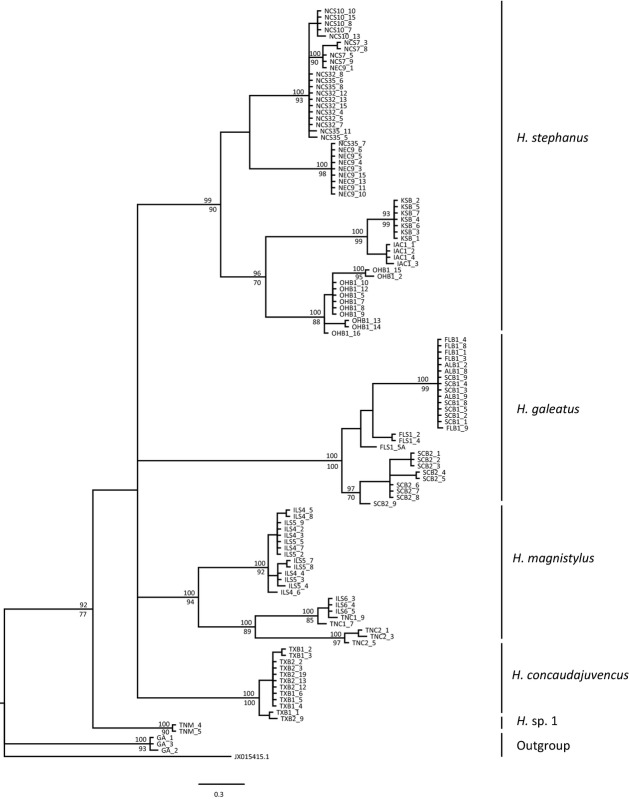
Molecular phylogeny of *Hoplolaimus* species based on the COI region; 50% majority consensus tree obtained with MrBayes (GTR + I + G model). Node-support values: upper value posterior probability BI shown if >95%, lower value bootstrap from ML analysis shown only if >70%. Subgenus *Hoplolaimus* as defined by (Siddiqi 2000) is supported. Out-group GA_1, _2 and _3; populations of *H. columbus* are representatives of subgenus *Basirolaimus*.

Phylogenetic reconstruction using the mtDNA sequences with ML and BI yielded trees with highly similar topologies and revealed five major clades (Fig.2). A first strongly supported clade (ML bootstrap 100%, BI posterior probability 1) comprised all specimens morphologically assigned to *H. galeatus*. Specimens from this clade were collected from Alabama (AL), Florida (FL), and South Carolina (SC), on bermudagrass, St. Augustinegrass, and bentgrass, respectively. A second strongly supported clade (ML bootstrap 94. %, BI posterior probability 1) included specimens identified as *H. magnistylus* and was collected from Illinois (IL) and two localities in Tennessee (TN) on soybean (*G. max*) and corn (*Z. mays*). A third clade (ML bootstrap 90%, BI posterior probability 0.99) comprised populations of *H. stephanus* from Nebraska (NE), Kansas (KS), Iowa (IA), Ohio (OH), and North Carolina (NC) collected on corn, bentgrass, and soybean. A fourth clade (ML bootstrap 99.9%, BI posterior probability of 1) was composed of individuals identified by morphology as *H. concaudajuvencus* and collected on bentgrass in Texas. Lastly, two specimens collected from maple tree (*Acer* sp.) in TN and classified according to morphological traits as *H*. sp. 1 (under description) formed a fifth clade (ML bootstrap 77%, BI posterior probability 0.92) sister to all remaining clades. The NJ analysis was performed only with the COI dataset and revealed a topology mostly congruent with the ML and BI trees, with similar sequence segregation within clades. The main difference with the other trees was that the *H*. sp. 1 clade was placed as a sister lineage of the out-group, *H. columbus*.

**Figure 3:**
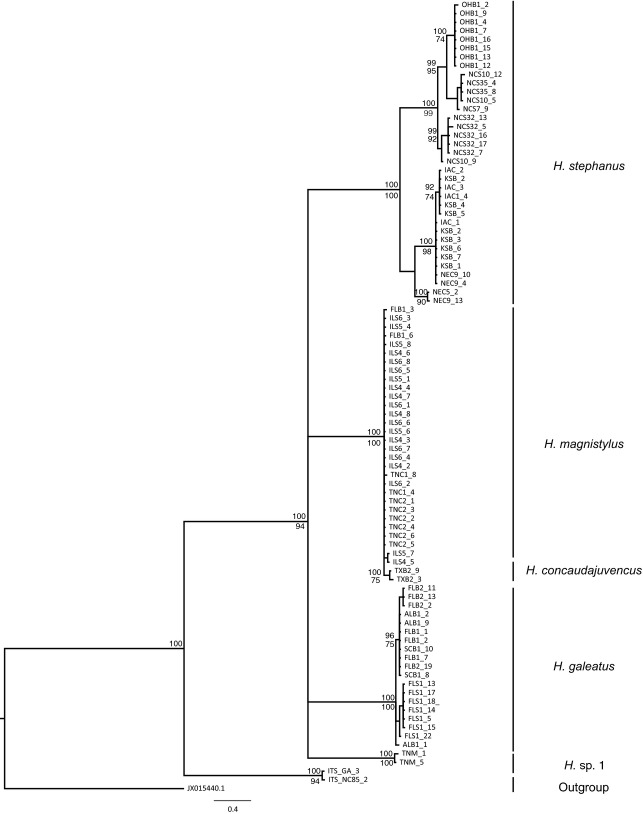
Molecular phylogeny of *Hoplolaimus* species based on the ITS region; 50% majority consensus tree obtained with MrBayes (GTR + I + G model). Node-support values: upper value posterior probability BI shown if >95%, lower value bootstrap from ML analysis shown only if >70%.

Within clades, phylogenetic analyses using the three methods (ML, BI, and NJ) for the COI region showed that *Hoplolaimus* individuals were structured according to locality (Figs.2, 3). In the *H. stephanus* clade, for example, COI sequences were subdivided into two major lineages: one composed mainly of different populations from NC and a sister clade mostly comprised of individuals from NE. The other *H. stephanus* subclade included three geographically clear lineages: individuals from IA that formed a single lineage closely related to the KS population and a third distinct lineage corresponding to populations from OH. Similar topology was revealed within the *H. magnistylus* clade, with a single clear subclade formed by two populations from IL and another lineage mostly composed of TN populations. Specimens of *H. concaudajuvencus* were only collected on grasses from Texas. The *H. galeatus* clade was the only one that did not show geographic structure, with two major subclades with broadly overlapping geographic ranges, which included populations from FL, SC, and AL.

For the nuclear marker, the ITS1 trees using ML and BI methods identified similar clades as for the COI sequences, with the difference that *H. concaudajuvencus* individuals were included in the *H. magnistylus* clade (Fig.3), and trees estimated from nuclear data gave less resolution, with little or no structure within clades. The only clade that showed fragmentation with ITS1 was *H. stephanus*, in which IA, KS, and NE populations formed one subclade, and OH and NC populations appeared as a closely related group (Fig.3). In general, the BI tree for ITS1 was supported by strong posterior probabilities (0.9–1), whereas the ML tree had bootstrap values from 35 to 100%. Another difference was that sequences from *H*. sp. 1 appeared as a sister clade of *H. galeatus* although with low bootstrap support (37%) (Fig.3).

Genetic distances (K2P) for COI between *Hoplolaimus* groups (clades) ranged from 11.65% to 23.21% with a mean of 16.64%. The maximum interspecific distances (22.78% and 23.21%) were found between *H. galeatus* and *H. columbus* populations, and the minimum values (11.65% and 11.99%) were found between *H. stephanus* and *H. magnistylus* populations from NC and IL, and OH and IL. Within groups (clades), genetic divergence varied from 0 to 12.83% with an average of 6.26%. Therefore, a clear overlap between intra- and interspecific genetic distances was detected. The deepest levels of intragroup divergence that caused this overlap mainly occurred between populations of the *H. stephanus* clade: OH and NE (10.93–12.83%), NC and IA (10.55–12.83%), NC and KS (11.31–12.44%), and NE and KS (11.29–12.44%). Additional high levels of intraspecific genetic divergence (10.91–12.04%) occurred in the *H. magnistylus* clade between some populations from IL and TN. In general, the *H. galeatus* clade showed lower levels of intraspecific divergence ranging from 0 to 7.01%.

### COI mtDNA population structure

**Figure 4:**
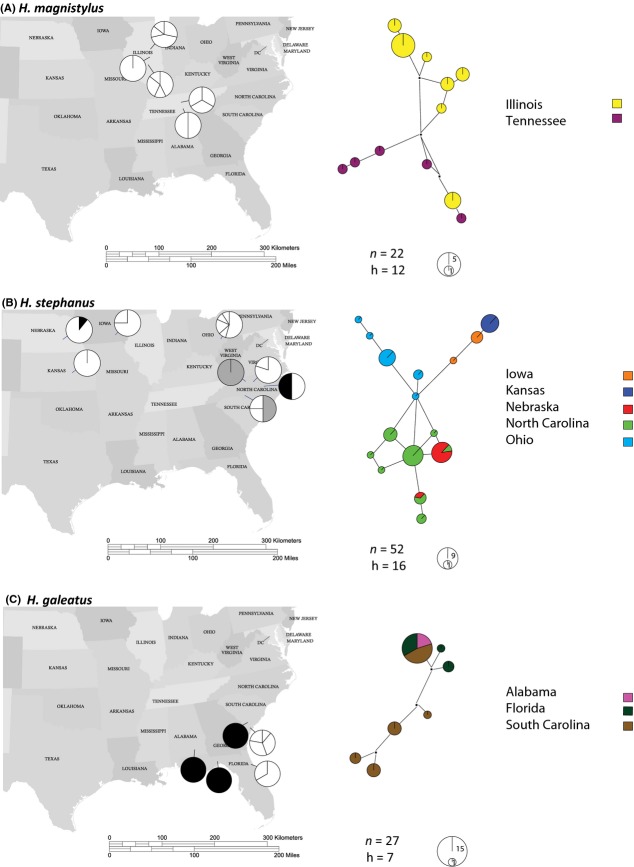
Distribution of the COI haplotypes and haplotype networks for the most predominant *Hoplolaimus* species found in this study, (A) *H. magnistylus,* (B) *H. stephanus, and* (C) *H. galeatus. n*: number of individuals and h: number of haplotypes. The size of each circle in the haplotype network is proportional to the number of individuals sharing the same haplotype. White indicates unique haplotypes; black and gray shadings indicate shared haplotypes.

Haplotype networks for COI were congruent with the phylogenetic reconstructions, showing segregation of the mitochondrial haplotypes according to locality (Fig.4). Sixteen haplotypes were identified in the *H. stephanus* clade, distributed in three main geographic groups: one mostly composed of populations from NC and NE, which share two haplotypes among them; the second with unique haplotypes from OH populations; and the third comprised of two haplotypes from IA and one from KS populations. In the *H. magnistylus* network*,* the 12 haplotypes identified were divided into two groups: one composed of haplotypes from populations collected in IL, and the second of haplotypes from TN populations. For *H. concaudajuvencus* and *H. galeatus,* fewer haplotypes were identified (4 and 7, respectively). For *H. concaudajuvencus*, all were unique haplotypes from TX populations. *Hoplolaimus galeatus* was the only species that did not show correlation with geographic regions, having a single haplotype shared by several individuals from SC, AL, and FL, with the remaining haplotypes being from populations collected in SC.

Frequencies and distribution patterns of COI haplotypes showed that *H. stephanus* was the most widespread and with the most diverse lineage, distributed from NE, KS, IA, and OH to NC (Fig.4). *Hoplolaimus galeatus* was the least diverse species with a predominant haplotype present in populations from AL, one from FL and one from SC. Haplotypes for the remaining species were predominantly location specific, conforming haplogroups concordant with sampling locations. The highest nuclear diversity was observed in *H. stephanus* (*π*= 0.068) and *H. magnistylus* (*π*=0.052). Haplotype diversity (Hd) was similar for *H. stephanus* and *H. magnistylus* (0.908 and 0.909, respectively) (Table2 and Fig.4).

Results from the 112 haplotypes analyzed with AMOVA suggest strong genetic differentiation in all species (clades) as explained by the high F_ST_ value (0.96%, *P *<* *0.0001) (Table3). AMOVA also revealed that 51.11% of the mtDNA variation was attributed to differences among the *Hoplolaimus* clades, followed by the variation among populations within each clade (43.07%) and very low variation within populations (5.82%). However, higher variation (76.79%) was recovered when AMOVA was performed across geographic regions, suggesting strong genetic differentiation among sampling locations. With regard to each morphospecies, the strongest genetic subdivision was found within the *H. stephanus* populations (85.11%), whereas populations of *H. galeatus* and *H. magnistylus* were less differentiated. The strong genetic differentiation in *H. stephanus* and the high intraspecific variability caused overlap between intra- and interspecific K2P genetic distances. Therefore, AMOVA was performed for *H. stephanus* with different combinations of populations to determine which grouping better explained the variability among groups. Results from the AMOVA showed the highest variation (83.10%) when populations of *H. stephanus* were partitioned as NC, NE, OH, and KS+IA. Based on this combination of populations, new K2P genetic distances were estimated considering these populations as different genetic entities (interspecific variability), and a barcoding gap was detected for this species.

**Table 3:** Sample sizes (*n*), number of haplotypes (h), gene diversity (Hd), and nucleotide diversity (*π*) for the four predominant *Hoplolaimus* species found in this study

*Hoplolaimus* species	*N*	h	Hd	*π*
*H. galeatus*	27	7	0.68	0.0300
*H. magnistylus*	22	12	0.91	0.0500
*H. stephanus*	52	16	0.91	0.0700
*H. concaudajuvencus*	12	4	0.56	0.0030

**Table 4:** Results of AMOVA analysis of cytochrome c oxidase subunit I (COI) for all *Hoplolaimus* individuals, within each *Hoplolaimus* species and for different partitions for *H. stephanus* populations. Number of individuals analyzed (*n*), proportion of variance explained (%), fixation index (ΦST), and the significant level ^*^^*^^*^P <* *0.001

	*n*	%	ΦST	*P*
*All sequences*	101		0.96	^*^^*^^*^
Among populations		76.79		
Within populations		18.98		
*H. galeatus*	27		0.8	^*^^*^^*^
Among populations		48.1		
Within populations		40.4		
*H. magnistylus*	22		0.94	^*^^*^^*^
Among populations		39.6		
Within populations		54.84		
*H. stephanus*	52		0.9	^*^^*^^*^
Among populations		85.1		
Within populations		4.95		
(NC, NE)(OH, KS, IA)			0.91	^*^^*^^*^
Among populations		63.55		
Within populations		27.49		
(NE)(NC)(KS, IA)(OH)			0.9	^*^^*^^*^
Among populations		82.09		
Within populations		8.03		

## Discussion

Molecular studies on *Hoplolaimus* species have resolved some phylogenetic relationships within the genus using the internal transcribed spacer (ITS1) (Bae et al. 2008), the 28S ribosomal DNA (Bae et al. 2009), and the actin gene (Ma et al. 2011). Mitochondrial markers had not been used before for molecular analysis of this genus. The protein coding cytochrome oxidase 1 (COI) gene has been proposed for species delimitation in several taxa by comparing pairwise genetic divergence among individuals of the same species versus individuals of different species (Hebert et al. 2003). Although the reliability of genetic distances for species delimitation has been subject of criticism (De Ley et al. 2005; DeSalle et al. 2005), COI barcoding can be a useful tool that complements morphological and molecular identification methods in an integrated taxonomy approach (Ferri et al. 2009).

In the COI haplotype distribution map, *Hoplolaimus* species and populations appear allopatrically distributed (geographically isolated). Genetic divergence between allopatric populations depends on the timing and level of gene flow between them (Mayr 1963; Nosil 2008). Geographic barriers seem to be the main source of variation for nematodes and soil-dwelling arthropods due to the low dispersal capacity of nematodes in soils, causing high levels of population fragmentation, which eventually lead to diversification of the species (Picard et al. 2004). Low gene flow among populations may have influenced the deep structure detected in the COI sequences. However, several authors have detected high gene flow over large distances in plant-parasitic nematodes such as *H. schachtii, G. pallida,* and *Bursaphelenchus mucronatus* (Picard et al. 2004; Plantard and Porte 2004; Pereira et al. 2013). For *C. elegans*, a free-living soil nematode, long-range dispersal was also suggested (Koch et al. 2000). For *H. schachtii*, *G. pallida,* and *B. mucronatus,* the high gene flow was attributed to transport of soil by farm machinery, sewage farms around sugar factories, by water through irrigation, flood or drainage, and /or by wind. For *H. galeatus*, we hypothesize that the application of nematicides could be responsible for the reduction in genetic diversity within populations, considering that this species was identified mainly on golf courses that require high maintenance and nematicide applications.

*Hoplolaimus stephanus* showed deep intraspecific variation that might be an indication of cryptic speciation. Strong population structure and genetic differentiation interpreted as cryptic speciation has been identified for marine and free-living nematodes using the same set of mitochondrial primers used in this study (Derycke et al. 2005; Ristau et al., 2013). The mitochondrial data evidenced the presence of three main groups in the *H. stephanus* haplotype network (NC + NE, OH, KS + IA). These groups also showed large K2P genetic distances that overlapped with the interspecific genetic distances, and AMOVA was better explained when the populations were partitioned in the same groups. However, the fragmentation in the nuclear marker for *H. stephanus* was not consistent with the one for COI, ITS1 gene partitioned IA, KS and NE populations in one group, and OH and NC populations composed the other group. Therefore, additional morphological evidence and molecular markers should be considered to confirm cryptic speciation for *H. stephanus*. Because almost all sampling localities of *H. stephanus* had their private haplotypes, it is likely that unsampled regions will also have their own lineages. If so, we are confronted with a multitude of lineages which are indistinguishable based on morphology and difficult to integrate into the framework of classical taxonomy. One possibility is to interpret *H. stephanus* as a polytypic species with extreme population differentiation.

This study is the first to use molecular phylogenetic analysis based on mitochondrial data for species within the genus *Hoplolaimus*. The COI gene resolved species in highly supported clades and showed deep variation within species, mainly correlated with geographic origin of the populations, indicating that this portion of the COI gene is adequate to define species and to study the genetic structure. Furthermore, we observed no heteroplasmy (presence of more than one mitochondrial genome in an individual) as evidenced by clean chromatograms with the absence of double peaks. Heteroplasmy has been reported in plant-parasitic nematodes like *M. chitwoodi* (Humphreys-Pereira and Elling 2013). However, Kiewnick et al. (2014) studied the feasibility of mitochondrial markers COI and COII on several *Meloidogyne* populations and did not detect heteroplasmy.

With ITS sequences, high genetic structure was only detected for *H. stephanus*. Previous studies on the phylogenetic relationships of *Hoplolaimus* species (Bae et al. 2008, 2009) found that the intron ITS1 showed higher variability among species within this genus. Our data showed that ITS1 provided poor resolution within clades, showing low variability for the majority of lineages. Other studies on the genetic diversity of plant-parasitic nematodes such as reniform nematode also detected a lack of genetic differentiation in the ITS region (Agudelo et al. 2005) and recently, Fu et al. (2011) for species discrimination within the *Heterodera* genus. This confirms the need to use different markers to infer phylogenetic relationships and for genetic population structure analysis. Incomplete lineage sorting could be a possible explanation for the high level of mitochondrial differentiation detected compared to the nuclear sequences (Gebiola et al. 2012), which is an expected consequence of populations undergoing speciation. The end result within a phylogeny is a subset of characteristics that have discordance within the species tree (Derycke et al. 2005).

It is possible that the inconsistency between nuclear and mitochondrial differentiation is attributable to a higher rate of molecular evolution for the latter. Because haploidy and uniparental inheritance reduce the effective number of mitochondrial genes to about one quarter of that of nuclear (Piganeau and Eyre-Walker, 2009), divergence can be accomplished earlier than for nuclear genes after a recent speciation event. Alternatively, the failure to detect nuclear DNA variation could be an artifact of the use of an inappropriate marker, although ITS rRNA markers have proven useful for species diagnosis in many nematode taxa (De Ley et al. 2005). The primers used in this study were also tested by Toumi et al. (2013) for discriminating several *Heterodera* and *Punctodera* species with successful results, which suggest that this portion of the COI gene can be a suitable marker for plant-parasitic nematode species. A nuclear marker that could be considered in future analysis is elongation factor-1*α* (EF-1*α*), a conserved nuclear coding gene that can be used to investigate recent divergences due to the presence of rapidly evolving introns (Kawakita et al. 2003). An ideal marker to confirm the lack of congruence between nuclear and mitochondrial differentiation is microsatellites, which are more sensitive in detecting recent or ongoing speciation events (Michel et al. 2010).

Morphological and molecular data using nuclear and mitochondrial genes revealed the presence of four recognized *Hoplolaimus* morphospecies in the soil samples collected: *H. galeatus, H. magnistylus, H. concaudajuvencus,* and *H. stephanus*. A fifth species (*H*. sp. 1), currently undescribed, was collected on maple. This is the first report for *H. concaudajuvencus* in TX on bentgrass and for *H. magnistylus* on corn in IL. Ours is also the first report for *H. stephanus* in OH and KSon bentgrass, and in IO and NE on corn. Our group had recently published a first report of *H. magnistylus* in TN on soybean, corn, and cotton (Donald et al. 2013).

To our surprise, *H. galeatus,* the species most frequently reported in crops in the United States, was only identified in SC, FL, and AL on turfgrasses. This species also exhibited low genetic diversity (low haplotype and nucleotide diversity) and showed the shortest genetic distances among its populations. In contrast, *H. stephanus*, the species with the fewest reports from agricultural soils, was the most common and diverse species found in this study. Sher (1963) described *H. stephanus* from specimens collected in Nicols, SC, and in the description included mention of an additional population from New Jersey. Nearly three decades later, Vovlas et al. (1991) published morphological observations from individuals collected in Raleigh, NC, from an unspecified host. Two decades later, Ma et al. (2011) found three populations of *H. stephanus,* one in Pennsylvania and two in SC from which they published nuclear sequences and designed species-specific primers for molecular diagnosis. The population from Pennsylvania (Ma et al. 2011) constitutes the only report of this species on a grass (*Poa pratensis*) host. These three publications total the extent of the research available for *H. stephanus*. Considering it appears to be the most widely distributed lance nematode species in agricultural soils in the United States, further work is needed to study its biology, ecology, pathogenicity, and economic thresholds.

## References

[b1] Agudelo P, Robert TR, Stewart JM, Szalanski AL (2005). Intraspecific variability of *Rotylenchulus reniformis* from cotton-growing regions in the United States. J. Nematol.

[b2] Alfaro ME, Zoller S, Lutzoni F (2003). Bayes or bootstrap? A simulation study comparing the performance of bayesian markov chain Monte Carlo sampling and bootstrapping in assessing phylogenetic confidence. Mol. Biol. Evol.

[b3] Bae CH, Szalanski AL, Robbins RT (2008). Molecular analysis of the lance nematode, *Hoplolaimus* spp., using the first internal transcribed spacer and the D2-D3 expansion segments of 28S ribosomal DNA. J. Nematol.

[b4] Bae CH, Robbins RT, Szalanski AL (2009). Molecular identification of some *Hoplolaimus* species from the USA based on duplex PCR, multiplex PCR and PCR-RFLP analysis. Nematology.

[b5] Baeza JA, Fuentes S (2013). Exploring phylogenetic informativeness and nuclear copies of mitochondrial DNA (numts) in three commonly used mitochondrial genes: mitochondrial phylogeny of peppermint, cleaner, and semi-terrestrial shrimps (Caridea: Lysmata, Exhippolysmata, and Merguia). Zool. J. Linnean Soc.

[b7] Castresana J (2000). Selection of conserved blocks from multiple alignments for their use in phylogenetic analysis. Mol. Biol. Evo.

[b8] De Ley P, De Ley IT, Morris K, Abebe E, Mundo-Ocampo M, Yoder M (2005). An integrated approach to fast and informative morphological vouchering of nematodes for applications in molecular barcoding. Philos. T. R. Soc. B.

[b9] De Oliveira DAS, Decraemer W, Holovachov O, Burr J, De Ley IT, De Ley P (2012). An integrative approach to characterize cryptic species in the *Thoracostoma trachygaster* Hope, 1967 complex (Nematode: Leptosomatidae). Zool. J. Linnean Soc.

[b10] Derycke S, Remerie T, Vierstraete A, Backeljau T, Vanfleteren J, Vincx M (2005). Mitochondrial DNA variation and cryptic speciation within the free-living marine nematode *Pellioditis marina*. Mar. Ecol-Prog. Ser.

[b11] Derycke S, Backeljau T, Vlaeminck C, Vierstraete A, van Fleteren J, Vincx M (2007). Spatiotemporal analysis of population genetic structure in *Geomonhystera disjuncta* (Nematoda, Monhysteridae) reveals high levels of molecular diversity. Mar. Biol.

[b12] Derycke S, Vanaverbeke J, Rigaux A, Backeljau T, Moens T (2010). Exploring the use of cytochrome oxidase c subunit 1 (COI) for DNA barcoding of free-living marine nematodes. PLoS ONE.

[b13] DeSalle R, Egan MG, Siddall M (2005). The unholy trinity: taxonomy, species delimitation and DNA barcoding. Philos. T. R. Soc. B.

[b14] Donald P, Holguin CM, Agudelo P (2013). First report of Lance nematode (*Hoplolaimus magnistylus*) on corn, soybean and cotton in Tennessee. Plant Dis.

[b15] Excoffier L, Laval G, Schneider S (2005). Arlequin ver. 3.0: An integrated software package for population genetics data analysis. Evol. Bioinf. Online.

[b511] Fassuliotis G (1974). Host range of the Columbia lance nematode *Hoplolaimus columbus*. Plant Dis. Rep.

[b16] Felsenstein J (1985). Confidence limits on phylogenies: an approach using the bootstrap. Evolution.

[b17] Ferri E, Barbuto M, Bain O, Galimberti A, Uni S, Guerrero R (2009). Integrated taxonomy: traditional approach and DNA barcoding for the identification of filarioid worms and related parasites (Nematoda). Front. Zool.

[b18] Fortuner R, Nickle WR (1991). The Hoplolaiminae. Manual of agricultural nematology.

[b522] Fu B, Yuan HX, Zhang Y, Hou XS, Nian GL, Zhang P (2011). Molecular characterisation of cereal cyst nematodes in winter wheat on the Huang-Huai floodplain of China using RFLP and rDNA-ITS sequence analyses. Australas. Plant Pathol.

[b19] Gangolf J, von Haeseler A, Strimmer K (2004). TREEFINDER: A powerful graphical analysis environment for molecular phylogenetics. BMC Evol. Biol.

[b20] Gebiola M, Gómez-Zurita J, Monti M, Navone P, Bernardo U (2012). Integration of molecular, ecological, morphological and endosymbiont data for species delimitation within the *Pnigalio soemius* complex (Hymenoptera: Eulophidae). Mol. Ecol.

[b21] Golden M, Minton N (1970). Description and Larval Heteromorphism of *Hoplolaimus concaudajuvencus*, n. sp. (Nematoda: Hoplolaimidae). J. Nematol.

[b22] Handoo Z, Golden AM (1992). A key and diagnostic compendium to the species of the genus *Hoplolaimus* Daday, 1905 (Nematoda: Hoplolaimidae). J. Nematol.

[b23] Hebert PDN, Cywinska A, Ball SL, deWaard JR (2003). Biological identifications through DNA barcodes. P. Roy. Soc. Lond. B. Bio.

[b24] Hebert PDN, Stoeckle MY, Zemlak TS, Francis CM (2004). Identification of birds through DNA barcodes. PLoS Biol.

[b533] Henn RA, Dunn RA (1989). Reproduction of *Hoplolaimus galeatus* and growth of seven St. Augustinegrass (*Stenotaphrum secundatum*) cultivars. Nematropica.

[b555] Hu M, Chilton NB, Zhu XQ, Gasser RB (2002). Single-strand conformation polymorphism-based analysis of mitochondrial cytochrome c oxidase subunit 1 reveals significant substructuring in hookworm populations. Electrophoresis.

[b26] Humphreys-Pereira DA, Elling AA (2013). Intraspecific variability and genetic structure in *Meloidogyne chitwoodi* from the USA. Nematology.

[b27] Jenkins WR (1964). A rapid centrifugal-flotation technique for separating nematodes from soil. Plant Dis.

[b28] Kawakita A, Sota T, Ascher JS, Ito M, Tanaka H, Kato M (2003). Evolution and phylogenetic utility of alignment gaps within intron sequences of three nuclear genes in Bumble bees (*Bombus*. Mol. Biol. Evol.

[b599] Kiewnick S, Holterman M, van den Elsen S, van Megen H, Frey J, Helder J (2014). Comparison of two short DNA barcoding loci (COI and COII) and two longer ribosomal DNA genes (SSU & LSU rRNA) for specimen identification among quarantine root-knot nematodes (Meloidogyne spp.) and their close relatives. Eur. J. Plant Pathol.

[b29] Koch R, van Luenen HGAM, van der Horst M, Thijssen KL, Plasterk RHA (2000). Single nucleotide polymorphisms in wild isolates of *Caenorhabditis elegans*. Genome Res.

[b30] Lewis SA, Riggs RD, Fassuliotis G (1982). Lance nematodes, *Hoplolaimus* spp., in the Southern United States. Nematology in the Southern region of the United States.

[b31] Librado P, Rozas J (2009). DnaSP v5: A software for comprehensive analysis of DNA polymorphism data. Bioinformatics.

[b32] Ma X, Agudelo P, Mueller JD, Knap HT (2011). Molecular characterization and phylogenetic analysis of *Hoplolaimus stephanus*. J. Nematol.

[b33] Mayr E (1963). Animal species and evolution.

[b34] Michel AP, Sim S, Powell THQ, Taylor MS, Nosil P, Feder JL (2010). Widespread genomic divergence during sympatric speciation. PNAS.

[b35] Moulton MJ, Song H, Whiting WF (2010). Assessing the effects of primer specificity on eliminating numt coamplification in DNA barcoding: a case study from Orthoptera (Arthropoda: Insecta). Mol. Ecol. Res.

[b610] Noe JP (1993). Damage functions and population-changes of *Hoplolaimus columbus* on cotton and soybean. J. Nematol.

[b36] Nosil P (2008). Speciation with gene flow could be common. Mol. Ecol.

[b611] Nyczepir AP, Lewis SA (1979). Relative tolerance of selected soybean cultivars to *Hoplolaimus columbus* and possible effects of soil temperature. J. Nematol.

[b37] Pereira F, Moreira C, Fonseca L, van Asch B, Mota M, Abrantes I (2013). New Insights into the phylogeny and worldwide dispersion of two closely related nematode species, *Bursaphelenchus xylophilus* and *Bursaphelenchus mucronatus*. PLoS ONE.

[b38] Picard D, Plantard O, Scurrah M, Mugniéry D (2004). Inbreeding and population structure of the potato cyst nematode (*Globodera pallida*) in its native area (Peru). Mol. Ecol.

[b39] Plantard O, Porte C (2004). Population genetic structure of the sugar beet cyst nematode *Heterodera schachtii*: a gonochoristic and amphimictic species with highly inbred but weakly differentiated populations. Mol. Ecol.

[b544] Piganeau G, Eyre-Walker A (2009). Evidence for variation in the effective population size of animal mitochondrial DNA. PLoS ONE.

[b40] Posada D, Crandall KA (1998). MODELTEST: Testing the model of DNA substitution. Bioinformatics.

[b566] Ristau K, Steinfartz S, Traunspurger W (2013). First evidence of cryptic species diversity and significant population structure in a widespread freshwater nematode morphospecies (*Tobrilus gracilis*. Mol. Ecol.

[b42] Robbins RT (1982). Description of *Hoplolaimus magnistylus* n. sp. (Nematoda: HopIolaimidae). J. Nematol.

[b577] Robbins RT (1987). Results of annual phytoparasitic nematode surveys of Arkansas soybean fields, 1978–1986. J. Nematol.

[b588] Robbins RT, Riggs RD, Von Steen D (1989). Phytoparasitic nematode surveys of arkansas wheat fields, 1986–88. J. Nematol.

[b43] Ronquist F, Huelsenbeck JP (2003). MRBAYES 3: Bayesian phylogenetic inference under mixed models. Bioinformatics.

[b44] Sher SA (1963). Revision of The Hoplolaiminae (Nematoda) II. *Hoplolaimus* Daday, 1905 and *Aorolaimus* N. Gen. Nematologica.

[b45] Siddiqi MR (2000). Tylenchida: parasites of plants and insects.

[b46] Song H, Buhay JE, Whiting MF, Crandall KA (2008). Many species in one: DNA barcoding overestimates the number of species when nuclear mitochondrial pseudogenes are coamplified. Proc. Natl Acad. Sci. USA.

[b47] Tamura K, Peterson D, Peterson N, Stecher G, Nei M, Kumar S (2011). MEGA5: Molecular evolutionary genetics analysis using maximum likelihood, evolutionary distance, and maximum parsimony methods. Mol. Biol. Evol.

[b48] Thompson JD, Gibson TJ, Plewniak F, Jeanmougin F, Higgins DG (1997). The ClustalX windows interface: flexible strategies for multiple sequence alignment aided by quality analysis tools. Nucleic Acids Res.

[b49] Toumi F, Waeyenberge L, Viaene N, Dababat A, Nicol JM, Ogbonnaya F (2013). Development of two species--specific primer sets to detect the cereal cyst nematodes Heterodera avenae and Heterodera filipjevi. Eur. J. Plant Pathol.

[b50] Vovlas N, Castillo P, Gomez Barcina A (1991). SEM observations on two species of Hoplolaimus Daday, 1905 (Nematoda: Hoplolaimidae). Nematol. Mediterr.

[b51] Zhang DX, Hewitt GM (1996). Nuclear integrations: challenges for mitochondrial DNA markers. Trends Ecol. Evol.

